# Mineral Particle Films as Climate-Change Adaptation Tools: Impacts on Growth, Yield, and Stress Mitigation in Grapevines

**DOI:** 10.3390/plants15152346

**Published:** 2026-07-30

**Authors:** Antonio Dattola, Gabriella Impallomeni, Beatrix Petrovicova, Rocco Zappia, Gregorio Gullo

**Affiliations:** Department of AGRARIA, Mediterranean University of Reggio Calabria, 89124 Reggio Calabria, Italy

**Keywords:** kaolin, grapevine, climate change, basalt powder

## Abstract

Viticulture is increasingly being threatened by the effects of climate change, particularly rising temperatures and prolonged drought, which can adversely affect vineyard management, productivity, and grape quality. To enhance vineyard resilience under Mediterranean conditions, this study evaluated the effectiveness of using two mineral particle films—calcined kaolin and basalt powder—as short-term adaptation tools for Nocera (*Vitis vinifera* L.) grapevines grown in the Faro DOC area (Sicily). Treatments were applied at key phenological stages to assess their capacity to mitigate thermal stress and preserve physiological activity during the hottest summer periods. Both formulations significantly improved yield components compared with untreated vines, primarily by reducing berry dehydration and maintaining bunch and berry mass. Treated vines also showed enhanced photosynthetic efficiency, higher stomatal conductance, and improved water use efficiency, indicating effective protection of the photosynthetic apparatus against heat- and light-induced stress. Must composition benefited from the treatments, demonstrating higher total soluble solids. The polyphenolic profile revealed treatment-specific metabolic responses: kaolin promoted higher flavanol and antioxidant accumulation, whereas basalt powder favored phenolic acids and UV-protective flavonols. Overall, mineral particle films proved to be valuable tools for sustaining productivity and grape quality in hot, dry seasons, supporting vineyard resilience without altering technological maturity.

## 1. Introduction

Agriculture is a fundamental pillar of the global economy, utilizing approximately 38% of the Earth’s land surface and nearly 70% of global freshwater resources for irrigation. However, climate change, combined with the projected world population growth to 9.7 billion by 2050, is expected to intensify pressure on agroecosystems [1]. Mediterranean regions are particularly vulnerable, as rising temperatures, altered precipitation patterns, and more frequent extreme events threaten crop productivity and long-term sustainability [2]. In viticulture, these climatic shifts disrupt the balance between climate, soil, and topography that governs vine physiology, grape composition, and wine quality. As a result, growers are increasingly adopting strategies that enhance vineyard resilience while maintaining production quality [3].

These adaptation measures include both long-term structural interventions, such as rootstock–scion combinations, planting density, and training systems, as well as short-term agronomic practices aimed at reducing leaf temperature and transpiration [4,5,6]. Among the latter, reflective mineral particle films have gained attention as sustainable tools to mitigate thermal and radiation stress. Several studies have demonstrated that particle film applications can reduce canopy temperature, improve light distribution, and enhance photosynthetic efficiency across multiple crops [7,8,9,10].

In viticulture, kaolin particle film (KPF) increases leaf and berry reflectance, thereby lowering surface temperature and improving the plant’s energy balance under high-radiation conditions. This cooling effect has been associated with improved photosystem II efficiency, higher photosynthetic capacity, and enhanced water use efficiency, in addition to reducing photoinhibition and oxidative stress. Kaolin applications have also been linked to improved grape quality, including higher acidity, lower pH, and increased phenolic content (particularly for anthocyanins) under hot and dry conditions [11]. Beyond its physical effects, kaolin influences hormonal regulation, especially ABA and IAA dynamics, and enhances antioxidant metabolism during summer stress, contributing to improved water relations, photosynthesis, and reduced oxidative damage [12,13,14]. It also stimulates phenyl-propanoid and flavonoid pathways, increasing phenolic and anthocyanin accumulation in berries [15].

In warm and arid climates, reflective films have been shown to improve berry coloration and increase the anthocyanin-to-soluble-solids ratio during ripening [16,17]. Similar effects have been reported for silicon-based foliar products, which can enhance stress tolerance and modulate secondary metabolism [18]. Other reflective minerals, such as basalt powder, have recently been investigated as alternatives or complements to kaolin. Petracca et al. demonstrated that basalt deposition on leaf surfaces does not interfere with gas exchange measurements or PSII photophysiology, confirming its suitability for ecophysiological studies [19] and supporting its practical use in vineyards to alleviate thermal and water stress under Mediterranean conditions [20,21,22].

Silica-based materials, including potassium silicate, have also been evaluated for their potential effects on grape composition. In a comparative study on Touriga Nacional and Touriga Franca (*Vitis vinifera* L.) grapes, kaolin tended to increase phenolic content, anthocyanins, tannins, and total phenols, highlighting its potential to preserve grape quality under climatic stress [23].

Within this framework, the present study aims to evaluate the effectiveness of two reflective mineral products—calcined kaolin, whose short-term mitigation effects against climate-related stress are well documented, and a newly formulated basalt powder—on the vegetative growth and yield performance of Nocera grapevines grown in the Faro DOC area of Sicily. By applying these treatments to the upper–middle canopy zone during key phenological stages, the study seeks to determine whether the basalt-based product can represent a viable or complementary alternative to kaolin under Mediterranean climatic conditions, particularly in relation to thermal stress reduction and improvements in vine performance.

## 2. Results

### 2.1. Effects of Treatments on Yield Components

The productive performance of Nocera grapevines was markedly influenced by the mineral particle-film treatments across both growing seasons, as confirmed by the biennial dataset presented in Table 1. Under drought and high-temperature conditions, the positive effects of kaolin (KT) and basalt powder (BPT) were primarily associated with enhancements in berry and bunch development rather than modifications in reproductive activity. This interpretation is supported by the absence of significant differences in the number of bunches per plant (BP) in either year (Table 1), indicating that yield improvements were driven by increases in berry and bunch mass rather than cluster number or bud fertility.

The yield per plant (YP) of treated vines was significantly higher in both 2024 and 2025 (Table 1). BPT consistently produced the greatest response, reaching 1.83 and 1.87 kg plant^−1^ in 2024 and 2025, respectively, corresponding to increases of approximately 20–21% compared with untreated vines (UT). KT also enhanced YP, though to a lesser extent (+8–9% across the two seasons). These results align with the higher hectare-scale productivity previously reported and confirm that both treatments improved fruit biomass accumulation under summer stress.

Significant differences in average rachis weight (ARW) were also detected, with a consistent pattern across years (Table 1). KT showed the highest ARW values (7.27 g in 2024 and 7.35 g in 2025), representing increases of about 70–75% relative to UT. BPT also induced significant improvements, though less pronounced. This suggests a greater effectiveness of kaolin in sustaining rachis development and structural functionality during berry growth and ripening.

Similarly, both treatments significantly increased average bunch weight (ABW) in each season (Table 1). KT and BPT produced heavier bunches, with increases of approximately 19–20% compared with UT, confirming the beneficial role of reflective particle films in supporting bunch development and maintaining berry turgor during late ripening. Average berry weight (ABeW) followed the same trend: BPT yielded the highest values in both years (2.75–2.79 g), corresponding to increases of about 20% relative to UT, while KT increased berry weight by approximately 14–15% (Table 1). These results support the hypothesis that both treatments mitigated dehydration and preserved berry water status under elevated thermal stress.

Overall, our biennial dataset clearly demonstrates that both kaolin and basalt powder improved the productive performance of grapevines under summer abiotic stress (Table 1). BPT was generally more effective in enhancing yield accumulation, whereas KT exerted a stronger influence on rachis development and tissue preservation, highlighting treatment-specific physiological responses.

### 2.2. Effects of Treatments on Grape Composition

The compositional characteristics of Nocera grapes at harvest were only partially influenced by the mineral particle-film treatments, as shown by the two-year dataset presented in Table 2. Among the measured parameters, total soluble solids (TSS) were the only variable consistently and significantly affected across both seasons. In each year, both kaolin (KT) and basalt powder (BPT) produced significantly higher TSS values than untreated vines (UT), confirming a treatment-related enhancement in sugar accumulation. BPT grapes showed the highest TSS in both 2024 (23.70 °Brix) and 2025 (23.66 °Brix), corresponding to an average increase of approximately 15% compared with UT (20.60–20.68 °Brix). KT also increased TSS, reaching 22.80–22.96 °Brix, equivalent to an improvement of about 10% relative to UT (Table 2). No significant differences were detected for pH in either season (Table 2).

Titratable acidity (TA) also did not differ significantly between treatments in either year. Although KT grapes exhibited numerically higher TA values (1.07–1.11%), these differences were not statistically significant and therefore represent trends rather than treatment-driven effects. TA values remained similar in BPT and UT grapes across both seasons, suggesting that the treatments did not substantially alter acid retention at harvest (Table 2).

The TSS/TA ratio showed no significant differences across treatments in either year, and the slightly higher values observed in BPT grapes should likewise be considered non-significant trends. The lower ratio observed in KT grapes was primarily attributable to their slightly higher acidity (Table 2). In addition to TSS, the treatments also influenced berry sugar content, calculated as the amount of soluble solids per berry. Across both seasons, BPT consistently produced the highest sugar content (651.80–659.10 mg berry^−1^), followed by KT (599.60–612.60 mg berry^−1^), whereas UT grapes showed markedly lower values (471.70–481.80 mg berry^−1^). These differences reflect the combined effect of higher TSS and greater berry mass in treated vines. The enhancement observed in BPT berries corresponds to an increase of approximately 35–40% relative to UT, while KT berries showed an improvement of about 25–30%.

Overall, our biennial dataset indicates that the mineral particle-film applications mainly improved grape technological maturity through enhanced sugar accumulation, while exerting limited and statistically non-significant influence on acidity-related parameters. Any numerical differences in pH, TA or TSS/TA should therefore be interpreted as non-significant trends rather than confirmed treatment effects.

### 2.3. Effects of Particle-Film Treatments on PSII Function

The photosynthetic performance of Nocera grapevines was markedly influenced by both mineral particle-film treatments, as demonstrated by the two-year dataset presented in Table 3. Significant differences were observed for all photochemical parameters except NPQ, and the absence of treatment × year interactions indicates that these responses were consistent across both seasons.

The maximum efficiency of photosystem II in light-adapted leaves (Fv’/Fm’) was significantly lower in untreated vines (UT), which reached values of 0.44–0.46, compared with both basalt powder (BPT) and kaolin (KT). Across the biennium, BPT leaves maintained values of 0.58–0.60, while KT leaves reached 0.61–0.63 (Table 3), corresponding to respective increases of approximately 30% and 38% relative to UT leaves. Although these values remained below those typically reported for non-stressed conditions, they clearly indicate improved PSII functionality in treated vines under drought and high-temperature stress.

A similar pattern was observed for the effective quantum yield of PSII (ΦPSII). UT plants consistently showed the lowest values (0.03–0.05), whereas BPT and KT plants reached 0.12–0.14 and 0.14–0.16, respectively (Table 3). Despite the large percentage increases, the key result is that both treatments significantly enhanced ΦPSII relative to UT in both years, confirming improved photochemical efficiency under stress. Photochemical quenching (qP) followed the same trend; UT plants exhibited the lowest values (0.12–0.14), while BPT and KT plants reached 0.22–0.24 and 0.24–0.26, respectively (Table 3). These increases correspond to improvements of approximately 77% for BPT and 92% for KT compared with UT, indicating a greater proportion of open PSII reaction centers in treated vines. In contrast, non-photochemical quenching (NPQ) did not differ significantly between treatments in either year, with values ranging from 1.93 to 2.32 (Table 3). This lack of variation suggests that the treatments did not substantially alter heat dissipation mechanisms. The electron transport rate (ETR) showed the clearest and most consistent treatment effect. UT resulted in the lowest values, whereas BPT and KT significantly increased the ETR to 97.5–99.8 and 111.0–113.7 µmol e^−^ m^−2^ s^−1^, respectively (Table 3). These values correspond to increases of approximately 47% for BPT and 67% for KT relative to UT, with KT exceeding BPT by about 14% across both seasons. Overall, our biennial dataset demonstrates that mineral particle-film applications substantially improved PSII photochemical performance under summer abiotic stress. While both treatments enhanced photosynthetic efficiency, KT consistently produced the strongest response across parameters, indicating a greater capacity to sustain electron transport and maintain PSII functionality under high-temperature and drought conditions.

### 2.4. Photosynthetic Efficiency and Leaf Microclimate Under Foliar Treatments

The data presented in Table 4 clearly demonstrate that both foliar treatments, basalt powder (BPT) and kaolin (KT), substantially improved the physiological performance of leaves relative to the untreated control (UT). The most pronounced enhancement was observed in net photosynthesis (An), where treated vines consistently exhibited higher assimilation rates across both seasons. In 2024, An increased from 5.58 µmol CO_2_ m^−2^ s^−1^ in UT plants to 7.82 and 8.14 µmol CO_2_ m^−2^ s^−1^ in BPT and KT plants, respectively, corresponding to improvements of approximately 39–45%. These trends were confirmed in 2025, indicating a stable and reproducible treatment effect across years. This sustained enhancement in carbon assimilation suggests that both treatments effectively mitigated leaf-level stress and improved photosynthetic functioning, likely through improved leaf microclimate or enhanced photoprotection mechanisms, as is frequently reported for kaolin films and mineral-based foliar sprays.

Stomatal conductance (gs) followed a similar pattern, with treated vines showing more than a twofold increase compared with UT. Although the percentage increase exceeded 100%, only qualitative differences are reported in this manuscript; nonetheless, the magnitude of this response underscores a substantial improvement in stomatal regulation under both treatments. The concurrent rise in An and gs suggests that treated leaves maintained more favorable gas exchange conditions, supporting higher CO_2_ uptake without incurring excessive water loss.

Despite these changes, transpiration rate (Tr) did not differ significantly among treatments in either year, as indicated by the “ns” notation in Table 4. This stability in Tr, combined with the marked increases in An, resulted in significantly improved water use efficiency (WUE) in treated vines. KT, in particular, showed the highest WUE values (3.74–3.78 µmol CO_2_ mmol^−1^ H_2_O), representing an improvement of approximately 35% over UT. This suggests that KT enhanced the plant’s capacity to assimilate carbon per unit of water transpired—a key advantage under conditions of elevated evaporative demand. The physiological implications of this pattern align with known benefits of reflective particle films in reducing leaf thermal load and improving stomatal behavior.

The An/Ci ratio, an indicator of carboxylation efficiency, also increased significantly under both treatments. BPT and KT vines demonstrated values of 0.032–0.035 compared with 0.024–0.026 in UT, corresponding to increases of 32–36%.

Similarly, SPAD values, reflecting chlorophyll content, were consistently higher in treated vines. KT vines showed the highest values (29.30–29.56), followed by BPT (28.50–28.76), while UT vines exhibited the lowest chlorophyll content (24.70–24.92). These increases of 15–19% relative to the UT group indicate that foliar treatments may have contributed to improved pigment stability or reduced photo-oxidative stress, consistent with their known protective roles.

**Figure 1:**
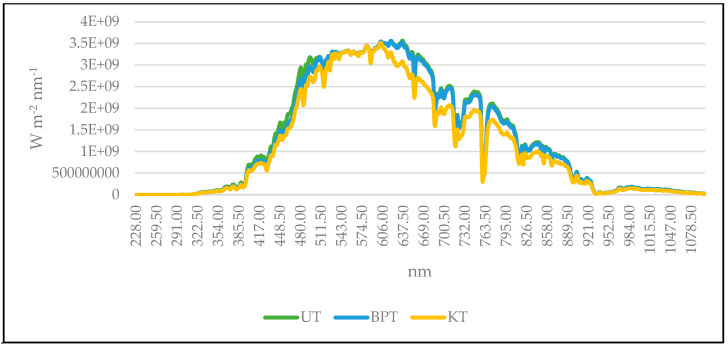
Spectral profiles recorded across the 300–1100 nm wavelength range. The curves represent the spectral irradiance expressed as W m^−2^ nm^−1^.

Leaf microclimate parameters further support these physiological trends. Treated vines exhibited lower leaf temperatures and reduced vapor pressure deficit (VPD) compared with UT, with KT showing the most favorable microclimatic conditions. For example, in 2024, leaf temperature decreased from 33.4 °C in UT vines to 31.2 °C in KT, while VPD decreased from 2.70 to 2.30 kPa. These modifications likely contributed to the improved gas exchange performance by reducing thermal and evaporative stress at the leaf surface. As shown in Figure 1, the spectral irradiance curves of the three treatments (UT, BPT, and KT) exhibited a similar overall pattern across the 300–1100 nm range, with a pronounced peak in the visible region. However, the KT treatment displayed consistently lower irradiance values in the near-infrared (NIR) region compared with the UT and BPT groups. The spectral profiles of UT and BPT largely overlapped, whereas KT vines showed a clear reduction in NIR intensity, indicating a distinct optical response associated with the kaolin treatment.

### 2.5. Stability and Selective Variation of Phenolic Compounds Under Different Foliar Treatments

Across the two growing seasons, the phenolic profile of Nocera berries (Table 5 and Table 6) showed that most compounds remained statistically unchanged between treatments, with only a limited subset exhibiting consistent differences. Among the hydroxybenzoic acids, gallic acid was the only molecule that responded to foliar applications, displaying higher concentrations in both KT and BPT plants compared with UT in 2024 (Table 5) and 2025 (Table 6). All other hydroxybenzoic acids, including protocatechuic, syringic, vanillic, and 2,5-dihydroxybenzoic acids, remained statistically unaffected in both years (Table 5 and Table 6). Among hydroxycinnamic acids, *p*-coumaric acid consistently showed the highest levels in UT plants, while KT and BPT markedly reduced its concentration in both 2024 (Table 5) and 2025 (Table 6). *o*-coumaric acid also differed significantly between treatments, reaching its highest values under KT in both seasons (Table 5 and Table 6), with UT and BPT plants showing intermediate and lower levels, respectively. In contrast, *m*-coumaric acid, trans-4-hydroxycinnamic acid, sinapic acid, 3-hydroxycinnamic acid, caffeic acid, and ferulic acid did not vary significantly in either year (Table 5 and Table 6). Flavan-3-ols exhibited the clearest treatment-related differences. Catechin and epicatechin were consistently higher in KT plants in both 2024 (Table 5) and 2025 (Table 6), whereas UT and BPT plants showed substantially lower concentrations. However, other compounds in the same class, such as procyanidin B2 and procyanidin 1, did not differ significantly in either season (Table 5 and Table 6), indicating that the response was restricted to specific molecules rather than representative of the entire flavan-3-ol group. Anthocyanidins, including pelargonidin and delphinidin, showed no significant variation between treatments in either 2024 (Table 5) or 2025 (Table 6). Similarly, most flavonols—such as quercetin, kaempferol, myricetin, rutin, and quercetin-3-β-D-glucoside—remained stable across treatments in both years (Table 5 and Table 6). Minor phenolics, including chlorogenic acid, vicenin-2, eriocitrin, tocopherol, ellagic acid, naringin, and apigenin, also showed no significant differences in either season (Table 5 and Table 6). Overall, our two-year dataset (Table 5 and Table 6) indicates that only a small number of phenolic compounds—specifically gallic acid, *p*-coumaric acid, *o*-coumaric acid, catechin, and epicatechin—showed consistent treatment-related variation across seasons, while the majority of the phenolic profile remained stable. These findings suggest that foliar applications of KT and BPT influenced a restricted subset of phenolic metabolites rather than inducing broad or coordinated changes in secondary metabolism.

## 3. Discussion

### 3.1. Effects of Kaolin and Basalt Powder on Vine Productivity

The application of kaolin (KT) and basalt powder (BPT) improved vine productivity under drought and high-temperature conditions [20]. Yield per vine increased by approximately 9% and 21% in KT and BPT vines, respectively, compared with untreated vines (UT) [21]. This increase was primarily attributable to greater cluster and berry weight, while the number of clusters per vine remained unchanged. The absence of significant differences in cluster number indicates that the yield enhancement in treated vines was mainly driven by increased berry growth and, consequently, greater cluster mass. These yield responses align with previous evidence showing that reflective particle films can support sink strength and carbohydrate allocation under stress, largely through improved photosynthesis and moderated canopy temperature.

### 3.2. Berry Growth and Thermal Stress Mitigation

The higher average berry weight observed in the KT group suggests that the treatment may have contributed to alleviating heat- and water-related stress during berry development. Because berry temperature was not directly measured, the present study cannot demonstrate that the treatment reduced berry temperature [10,13]. Instead, the observed response is consistent with the improved leaf physiological performance and lower leaf temperature recorded in treated vines, which may have indirectly contributed to maintaining berry water status. The maintenance of berry water status may derive from multiple physiological mechanisms. Lower berry temperature can directly reduce transpiration through the skin and moderate water-potential gradients between clusters and vegetative tissues. High temperatures are known to increase transpiration and promote water fluxes from ripening berries toward vegetative organs, contributing to reductions in their fresh weight during maturation [24]. Consequently, the higher berry weight may reflect a better physiological status of treated vines under summer stress. Similar findings have been reported in other studies involving kaolin and reflective materials applied to grapevines and Mediterranean fruit crops exposed to intense thermal and radiative stress [25,26,27,28,29,30]. The greater berry and cluster weight observed in treated vines is therefore consistent with improved plant water status and reduced sunburn damage under stressful environmental conditions [21]. These integrated responses suggest that reflective treatments may act simultaneously on leaf-level physiology and berry-level water balance, contributing to coordinated improvements in fruit growth.

### 3.3. Impacts on Must Composition

Only TSS and sugar content exhibited statistically significant differences among treatments within each year, indicating that the application of basalt powder and kaolin primarily affected the accumulation of soluble solids and the amount of sugars per berry. In contrast, pH, TA, and TSS/TA did not show significant variation across treatments, suggesting that these parameters remained stable regardless of the applied mineral particle films. To ensure full consistency between the statistical outputs and the interpretation of the results, letters indicating significant differences have been assigned exclusively to TSS and sugar content, which were the only variables for which Tukey’s post-hoc test identified treatment-related effects. This approach avoids implying significance where none was detected and aligns the presentation of the data with the outcomes of the two-way ANOVA. The discussion of technological maturity and acidity has also been revised accordingly. Although lower pH values in treated vines may be relevant under warm climate conditions—where excessive increases in must pH are commonly associated with accelerated ripening and loss of acidity [31,32,33,34,35], these observations are now presented as non-significant trends, consistent with the absence of statistical differences in titratable acidity. Similarly, references to the implications of pH for microbiological stability, color stability, and sensory freshness [36,37] have been retained but framed appropriately to avoid overinterpretation.

### 3.4. Photosynthetic Performance and Photoprotection

Physiological measurements showed that KT and BPT effectively mitigated the effects of high radiation and temperature on the photosynthetic apparatus. Treated vines maintained significantly higher values of Fv’/Fm’, ΦPSII, qP, and ETR compared with UT, indicating improved photosystem II efficiency and reduced photoinhibition. In particular, the increase in ETR (+47% in BPT and +67% in KT relative to UT) suggests a more efficient transfer of excitation energy along the electron-transport chain. These responses can be interpreted as a direct consequence of the reduction in radiative and thermal load at the leaf surface induced by reflective treatments. Kaolin and basalt powder increase the reflectance of incident radiation (UV, PAR, IR), thereby lowering leaf temperature and reducing excess energy absorbed by chloroplasts [38,39]. This effect is especially relevant under high-radiation and water-limiting conditions, where excess energy can compromise the stability of the oxygen-evolving complex and promote PSII inactivation. Under such conditions, reducing the energetic load allows for better regulation of energy-dissipation mechanisms, limiting the need for non-photochemical quenching [13,40]. At the same time, maintaining high photochemical efficiency favors the more effective use of electrons in carbon fixation, contributing to sustained net CO_2_ assimilation. The reduced NIR intensity observed in the KT treatment is consistent with the high reflectance of kaolin particle films, which decrease the absorption of infrared radiation by leaf tissues. From an ecophysiological perspective, limiting the interception of NIR—responsible for a substantial portion of leaf thermal load—may contribute to moderating leaf temperature under high-radiation conditions. A higher diffuse reflectance may also alter canopy light distribution, reducing localized hotspots. Overall, these physiological adjustments help explain the improvements observed in berry growth and yield, highlighting the tight linkage between photoprotection, carbon assimilation, and reproductive performance under Mediterranean summer stress. Importantly, the observed improvements in WUE and photosynthesis provide a mechanistic explanation for the increases in berry weight and yield, reinforcing the functional link between gas exchange dynamics and reproductive performance under stress.

### 3.5. Water Use Efficiency and Gas Exchange Dynamics

The improvement in photosynthetic activity was also reflected in greater water-use efficiency (WUE), which resulted from a combination of stomatal regulation and preserved PSII functionality, allowing vines to sustain higher photosynthetic rates without increasing water loss. KT-treated vines showed a significant increase in net CO_2_ assimilation (An) compared with the control, accompanied by more than doubled stomatal conductance (gs). However, this increase in stomatal opening did not lead to a corresponding rise in transpiration (E), which remained essentially unchanged. This can be attributed to the reflective effect of the materials, which reduces leaf temperature and the leaf-to-air vapor pressure deficit (VPD) [41]. Under these conditions, greater stomatal opening enhances CO_2_ uptake without proportionally increasing water loss. As a result, treated vines exhibited improved WUE, particularly those in the KT group (+35% relative to UT). Furthermore, the increase in the An/Ci ratio observed in treated vines suggests enhanced carboxylation efficiency and reduced biochemical limitations to photosynthesis. This indicates that improved photosynthetic performance was not solely due to greater stomatal CO_2_ availability but also to enhanced mesophyll capacity for carbon fixation, likely favored by the more favorable microclimatic conditions induced by the treatments [42,43]. The differences between KT and BPT may be associated with the distinct physical characteristics of the materials. While kaolin forms a highly reflective particle film, widely documented for its ability to modify canopy radiative balance, basalt powder acts primarily as a mineral dust deposited on the leaf surface, whose effects have been associated with the mitigation of environmental stress and improved ecophysiological performance, although the radiative mechanisms involved are less documented than those of kaolin [44]. This may explain the greater increases observed in ETR, An, catechin, epicatechin, and WUE in KT-treated vines. Conversely, the stronger yield response observed in BPT-treated vines suggests that additional physical or mineral effects may have contributed to plant performance, although the current data do not allow direct identification of these processes. Differences in particle size, adhesion, persistence on the leaf surface, and optical properties may have influenced canopy microclimate and physiological responses in different ways [45]. Similar physiological responses to reflective materials have been reported in other studies conducted on grapevines and Mediterranean fruit crops [10,21,41,46,47].

### 3.6. Modulation of Phenolic Metabolism

The phenolic profile revealed only a limited number of treatment-related differences, without evidence of broad or coordinated shifts in secondary metabolic pathways. Both KT and BPT resulted in significantly higher concentrations of gallic acid (approximately +29–35% compared with UT). Within the hydroxycinnamic acid group, untreated vines accumulated significantly higher levels of *p*-coumaric acid, whereas KT and BPT markedly reduced its concentration. A similar, though less pronounced, pattern was observed for *m*-coumaric acid, while *o*-coumaric acid reached its highest values under KT. KT also produced the clearest response among flavan-3-ols, with catechin and epicatechin concentrations significantly higher than in UT, although this trend was not reflected in other compounds of the same class. BPT showed slightly elevated levels of certain flavonols, such as myricetin and kaempferol, but these differences remained modest. Overall, phenolic responses were compound-specific and limited in magnitude, indicating that mineral particle films did not induce extensive modifications of grape secondary metabolism under the conditions of this study.

### 3.7. Overall Implications for Vine Resilience

Overall, the productive, physiological, and biochemical responses observed indicate that both kaolin and basalt powder improved the resilience of vines to the summer stress typical of Mediterranean environments. Improvements in photosynthetic performance, water-use efficiency, and berry growth were associated with specific modifications in phenolic metabolism, suggesting complementary stress-mitigation mechanisms. KT exerted stronger effects on gas exchange, flavanol accumulation, and antioxidant-related metabolites, whereas BPT showed greater influence on yield and the accumulation of selected flavonols. These findings highlight the potential of mineral reflective treatments as sustainable strategies to mitigate drought and heat stress in viticulture, contributing to the preservation of grape composition and technological quality under climate-change scenarios.

## 4. Materials and Methods

### 4.1. Pedoclimatic Context of the Study Area

**Figure 2:**
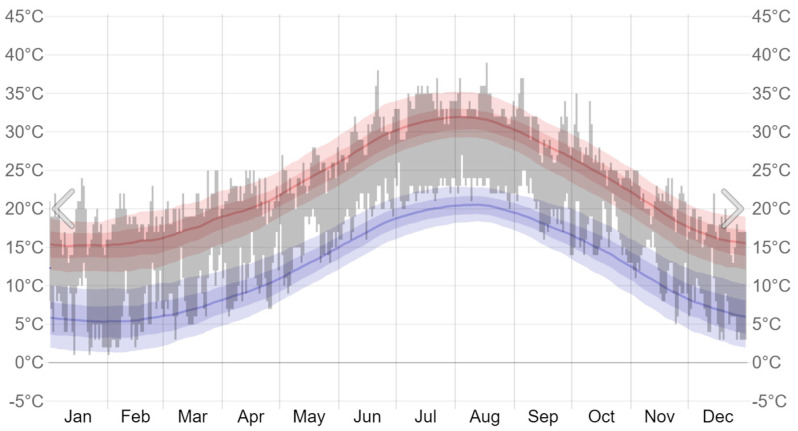
Thermal regime of the study area for the year 2024, illustrating the seasonal evolution of air temperature and the characteristic summer warming typical of the local Mediterranean climate. The colored bands represent different thermal ranges: the red band indicates higher temperatures, the grey band denotes average conditions, and the blue band corresponds to lower temperatures. Source: WeatherSpark (2024).

**Figure 3:**
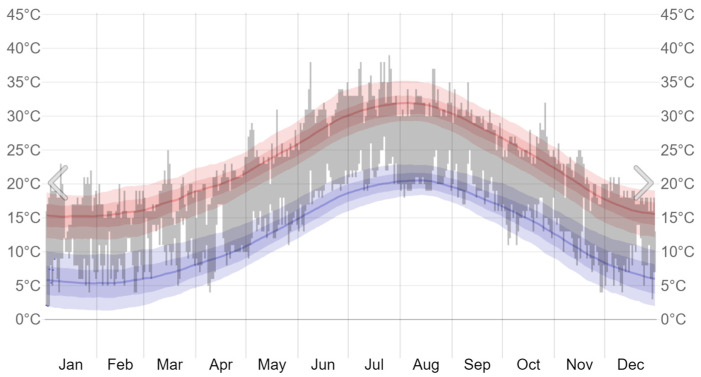
Thermal regime of the study area for the year 2025, illustrating the seasonal evolution of air temperature and the characteristic summer warming typical of the local Mediterranean climate. The colored bands represent different thermal ranges: the red band indicates higher temperatures, the grey band denotes average conditions, and the blue band corresponds to lower temperatures. Source: WeatherSpark (2025).

The pedoclimatic context of this experimental trial is a narrow coastal strip situated between the Tyrrhenian and Ionian Seas, within the Faro Controlled Designation of Origin (DOC). The production area lies entirely within the municipality of Messina, extending from Giampilieri Marina to Capo Peloro on the Ionian side and from Capo Peloro to Ortoliuzzo on the Tyrrhenian side. The study was conducted over the 2024–2025 period in a vineyard of *Vitis vinifera* L. cv. Nocera covering approximately 1 ha, established in 2005 and trained to a spur-pruned cordon system. Vine spacing was 2.50 m × 1.00 m, corresponding to a planting density of 4000 vines ha^−1^. The vines were trained on stakes positioned 80 cm above the ground, with 2 m above-ground supports. Winter pruning was carried out on two-bud spurs, maintaining a load of 12 buds per vine per linear meter of canopy wall. The experimental trials were performed on vines grafted onto rootstock 140 Ruggeri (140R). The vineyard is managed under rainfed conditions, with no supplemental irrigation applied during either season. Across both years, the temperature patterns reveal a climate which features strong seasonality and pronounced summer heat (Figure 2 and Figure 3). The July–August period consistently shows very high maximum temperatures (often above 35 °C) and elevated minima, indicating limited nocturnal cooling (Figure 2 and Figure 3). The widening gap between the upper and lower temperature ranges in summers suggests increased thermal variability and possible heatwave conditions, while winter values remain lower and more stable (Figure 2 and Figure 3). Weather station data showed typical Mediterranean rainfall patterns, with dry summers and high interannual variability. Total precipitation was 613.6 mm in 2024 and 802.0 mm in 2025, with rainfall concentrated mainly in late winter and autumn, while July remained extremely dry in both years (4–5 mm). Overall, the data depict an area characterized by intense, persistent summer warmth typical of a Mediterranean-type climate. The soil at the experimental site is classified as a clay loam—a medium-textured soil combining moderate porosity with substantial water-holding capacity. Field measurements were used to determine Field Capacity (30%) and Permanent Wilting Point (15%), while soil moisture was monitored using SM100 dielectric sensors (Delta-T Devices Ltd., Cambridge, UK).

### 4.2. Experimental Design

The experimental area was arranged in three randomized blocks, each containing three treatments—UT (untreated), KT (kaolin treatment), and BPT (basalt powder treatment)—with 12 vines per treatment per block, for a total of 36 vines per treatment. The shielding products were applied at three phenological stages corresponding to BBCH 75 (berry development), BBCH 77 (cluster closure), and BBCH 83 (veraison). During the 2024 growing season, applications were carried out on 27 June, 14 July, and 4 August, whereas in 2025 they were performed on 29 June, 16 July, and 6 August. The kaolin formulation (Serbios Srl, Rovigo, Italy) was applied at 5 kg hL^−1^, while Basalt Powder^®^ (Basalti Orvieto Srl, Terni, Italy) was applied at 3 kg hL^−1^. Treatments were applied using a backpack sprayer equipped with hollow cone nozzles operating at approximately 5 bar, calibrated to a flow rate of about 1.0 L min^−1^ (≈500 L ha^−1^). Both sides of the canopy were sprayed to ensure full coverage of leaves and clusters until near-runoff conditions were reached. A single pass per canopy side was performed for each application. Physiological measurements were conducted on vines from each treatment group following the applications. Grapes from each treatment were collected at technological maturity to ensure comparability among treatments, despite slight differences in the rate of ripening.

### 4.3. Eco-Physiological Measurements

Gas exchange and chlorophyll fluorescence parameters were measured on vines from each treatment. Measurements were taken on mature, fully expanded leaves located in the mid-canopy (3 leaves × 4 plants per treatment per block). Gas exchange determinations were conducted in the second half of August using a portable photosynthesis system (LI-6400XT; LI-COR Biosciences, Lincoln, NE, USA). Measurements were taken on a clear summer day at solar noon (12:00) under optimal atmospheric conditions. Leaf temperature was recorded using the thermocouple supplied with the system (model 6400-04). Chlorophyll content was estimated indirectly through SPAD readings using a SPAD-502 Chlorophyll Meter (Konica Minolta Inc., Tokyo, Japan), equipped with two optical sensors operating at different wavelengths and a silicon photodiode (SPD). A total of 36 leaves per treatment were measured. The spectral irradiance was measured at mid-day (12:00 h) using a spectroradiometer (Mod. PS-300 Spectroradiometer, Apogee Instruments Inc., Logan, UT, USA). Measurements were conducted using plexiglass plates treated with the same concentrations applied in the field in order to reproduce the optical conditions of the experimental treatments.

### 4.4. Morphobiometric and Qualitative–Quantitative Measurements

At harvest, for each vine within each treatment and block, yield per vine was determined in terms of both total weight and number of clusters. Three samples per treatment per block (one cluster per vine; three clusters from three vines) were transported to the Laboratory of Tree Crops of the Department of Agriculture, Mediterranean University of Reggio Calabria, where morphobiometric analyses and the determination of ripening indices were performed. Fresh weight (FW) of berries and clusters was measured using an analytical balance (BJ610C, Precisa Instruments AG, Dietikon, Switzerland). For each sample, 100 berries per treatment per block were stored at −80 °C for subsequent nutraceutical analyses, while another 100 berries were used for ripening assessments. Total soluble solids (TSS) were measured using a digital refractometer (mod. PAL-1, Atago Co., Ltd., Tokyo, Japan) and expressed as g soluble solids per 100 g must. Titratable acidity (TA) was measured using a potentiometric titrator (Titralab AT1000 series, HACH, Loveland, CO, USA); a 5 mL aliquot of must was titrated with 0.5 N NaOH until neutralization, and TA was expressed as % tartaric acid. The TSS/TA ratio was also calculated as an additional ripening index. For berry-related variables, 100 berries per treatment were sampled and treated as subsamples; individual measurements were averaged at vine level prior to statistical analysis. Sugar content per berry was estimated indirectly by combining the mean berry weight with the corresponding TSS value. Specifically, sugar content was calculated as mg of soluble solids per berry, obtained by multiplying berry fresh weight by TSS expressed as g soluble solids per 100 g must.

### 4.5. Determination of Total Phenolic Content and Phenolic Profiling by HPLC

Phenolic extraction and chromatographic analysis were performed using HPLC-grade solvents, including methanol and acetonitrile (Sigma-Aldrich, ≥99.99%), acetone (Sigma-Aldrich, ≥99.5%), deionized water, formic acid (Carlo Erba, 95%), and hydrochloric acid (Carlo Erba, 37%). A comprehensive set of analytical standards representing phenolic acids, flavonoids, anthocyanins, and tannins was obtained from Sigma Aldrich (Italy). Standards included gallic acid, protocatechuic acid, procyanidins B1 and B2, syringic acid, *p*, *m*, and *o*-coumaric acids, pelargonidin, trans cinnamic acid, bergamottin, cyanidin 3 O glucoside, catechin, vanillic acid, epicatechin, delphinidin, trans 4 hydroxycinnamic acid, sinapinic acid, 3 hydroxycinnamic acid, myricetin, luteolin, punicalagin, 2,5 dihydroxybenzoic acid, caffeic acid, ellagic acid, naringin, apigenin 7 neohesperidoside, spiraeoside, quercetin, kaempferol, tocopherol, chlorogenic acid, vicenin 2, eriocitrin, rutin, vitexin, quercetin 3 β D glucoside, ferulic acid, and apigenin. Standard solutions were prepared at four concentrations (0.01–0.10 mg mL^−1^) to generate external calibration curves. For phenolic profiling, berries were collected at harvest from clusters located in the middle canopy zone. To ensure representative sampling, berries were selected from the apical, median, and basal portions of each cluster. For each treatment, three independent biological replicates were obtained, each consisting of approximately 150 berries. The replicates were processed and analyzed separately without pooling; for each one, two technical HPLC replicates were performed to verify analytical repeatability. For sample preparation, 0.1 g of lyophilized grape skins was subjected to two extraction protocols: (i) extraction in 10 mL methanol acidified with 1% HCl, and (ii) extraction in 10 mL acetone (1% HCl) (1:1, *v*/*v*). Extracts were vortexed, centrifuged, filtered through 0.22 μm membrane filters, and prepared for chromatographic injection. Phenolic profiling was performed using a Thermo Scientific Dionex UltiMate 3000 HPLC system equipped with a diode array detector (DAD) and a Kinetex C18 column (250 × 4.6 mm, 5 μm). The column temperature was maintained at 30 °C. The mobile phase consisted of solvent A (water + 0.1% formic acid) and solvent B (acetonitrile + 0.1% formic acid), delivered at a flow rate of 1.0 mL min^−1^. The gradient elution program was as follows: 5% B at the initial condition, 15% B at 10 min, 25% B at 20 min, 40% B at 35 min, 60% B at 45 min, followed by re equilibration to the initial conditions. The injection volume was 10 μL. Detection was carried out at 260, 280, 300, and 520 nm, corresponding to the absorption maxima of the main phenolic groups. Compound identification was achieved by comparing retention times and UV–Vis spectra with those of authentic reference standards. Quantification was performed using external calibration curves, with coefficients of determination (R^2^) greater than 0.998 for all analytes. Method validation included determination of the limits of detection (LODs) and limits of quantification (LOQs) according to the 3σ and 10σ criteria, respectively. Intra and inter day repeatability were evaluated and showed coefficients of variation (CV) below 5%. Extraction recovery ranged from 88% to 104%, confirming satisfactory analytical accuracy and precision. All quantified phenolic compounds were subjected to statistical analysis using one way ANOVA followed by Tukey’s post hoc test (*p* < 0.05). In Table 5 and Table 6, significance letters are reported only for compounds showing statistically significant differences among treatments, whereas “ns” indicates the absence of significant differences.

### 4.6. Statistical Analysis

Statistical analyses were performed using SPSS v.22.0. A two-way analysis of variance (ANOVA) was used to evaluate the effects of year, treatment, and their interaction on all physiological, morphobiometric, and qualitative parameters. The experimental unit was the individual vine within a randomized complete block design. For each parameter, multiple subsample measurements (leaves, berries, clusters) were averaged at vine level prior to analysis to ensure biological replication and avoid pseudoreplication. This approach allowed assessment of whether variables were influenced independently by each factor or whether combined effects (year × treatment) contributed significantly to variability. When significant differences were detected, Tukey’s post hoc test was applied for mean separation (*p* ≤ 0.05).

## 5. Conclusions

This study demonstrates that mineral particle films, specifically calcined kaolin (KT) and basalt powder (BPT), can effectively mitigate the impacts of drought and high-temperature conditions in *Vitis vinifera* L. cv. Nocera cultivated in a Mediterranean environment. Across the two experimental seasons, both treatments enhanced vine performance compared with untreated plants, indicating an improved capacity to sustain physiological activity and productive efficiency under summer stress. Yield enhancement was primarily associated with increases in berry and cluster weight, whereas the number of clusters per vine remained unchanged, suggesting that the treatments influenced fruit growth and berry development rather than reproductive potential. Treated vines also exhibited higher total soluble solids than untreated vines, indicating a beneficial effect on grape technological maturity at harvest. Physiological measurements consistently revealed higher photosynthetic efficiency in treated vines, as reflected by improvements in chlorophyll fluorescence parameters, electron transport rate, net photosynthesis, stomatal conductance, and water-use efficiency. These responses indicate that both mineral films contributed to maintaining photosynthetic functionality under elevated thermal and radiative stress. Polyphenolic profiling revealed only limited treatment-dependent variation, with significant differences restricted to a small number of compounds. Although kaolin and basalt powder showed distinct tendencies in certain phenolic acids or flavonols, most phenolic constituents remained statistically unchanged across seasons. While must composition under KT and BPT suggests a potential technological suitability for Faro DOC wine production, these indications should be interpreted with caution. Because the study did not include vinification, wine chemical analyses, or sensory evaluation, the actual impact of mineral particle films on wine composition, sensory profile, and typicity remains to be determined and warrants further investigation. Overall, our results indicate that both KT and BPT can enhance grapevine resilience under Mediterranean summer conditions while maintaining yield performance and key grape quality attributes. The low environmental impact and practical applicability of these materials make them promising tools for climate-adaptation strategies in viticulture. Further investigations, including direct evaluations of the resulting wines, will help clarify the extent to which these treatments support or enhance the sensory and typicity requirements of the Faro DOC.

## Figures and Tables

**Table 1:** Yield components of Nocera cv. grapevines under different treatments (BPT = basalt powder; KT = kaolin; UT = untreated). YP = yield per plant (kg plant^−1^); ARW = average rachis weight (g); ABW = average bunch weight (g); ABeW = average berry weight (g); BP = number of bunches per plant. Values are expressed as mean ± standard deviation and refer to data collected over two growing seasons.

Year (Y)	Treatment (T)	YP kg Plant^−1^	ARW g	ABW g	ABeW g	BP n.
2024	BPT	1.83 ± 0.06 a	5.58 ± 0.04 b	262.1 ± 5.5 b	2.75 ± 0.02 a	7.10 ± 0.60 ns
	KT	1.66 ± 0.05 a	7.27 ± 0.05 a	260.0 ± 6.0 a	2.63 ± 0.03 a	5.90 ± 0.55 ns
	UT	1.52 ± 0.04 b	4.15 ± 0.10 c	219.0 ± 5.0 c	2.29 ± 0.03 b	5.15 ± 0.50 ns
2025	BPT	1.87 ± 0.07 a	5.66 ± 0.05 b	266.4 ± 6.2 b	2.79 ± 0.03 a	7.00 ± 0.70 ns
	KT	1.68 ± 0.04 a	7.35 ± 0.06 a	264.6 ± 5.8 a	2.67 ± 0.04 a	5.78 ± 0.60 ns
	UT	1.54 ± 0.05 b	4.29 ± 0.12 c	223.2 ± 5.3 c	2.33 ± 0.04 b	5.10 ± 0.55 ns
T		*	*	*	*	ns
Y		ns	ns	ns	ns	ns
T × Y		ns	ns	ns	ns	ns

Within each year, different letters indicate significant differences among treatments according to Tukey’s test (*p* < 0.05). The rows T, Y and T × Y refer to the overall effects of year, treatment and their interaction based on two-way ANOVA. * indicates a statistically significant effect (*p* < 0.05), whereas “ns” denotes a non-significant effect.

**Table 2:** Compositional characteristics of Nocera cv. grapes at harvest under the different treatments (BPT = basalt powder; KT = kaolin; UT = untreated). pH = must acidity level; TSS = total soluble solids (°Brix); TA = titratable acidity (% tartaric acid); TSS/TA = sugar-to-acid ratio, sugar content = soluble solids per berry (mg berry^−1^). Values are expressed as mean ± standard deviation and refer to data collected over two growing seasons.

Year (Y)	Treatment (T)	pH	TSS °Brix	TA %	TSS/TA	Sugar Content mg Berry^−1^
2024	BPT	3.18 ± 0.06 ns	23.70 ± 0.06 a	0.95 ± 0.05 ns	25.40 ± 2.20 ns	651.80 ± 19.42a
	KT	3.25 ± 0.14 ns	22.80 ± 0.38 a	1.07 ± 0.13 ns	21.80 ± 2.10 ns	599.60 ± 18.24 b
	UT	3.58 ± 0.20 ns	20.60 ± 0.40 b	0.90 ± 0.05 ns	23.00 ± 1.30 ns	471.70 ± 19.42 c
2025	BPT	3.22 ± 0.08 ns	23.66 ± 0.05 a	0.93 ± 0.05 ns	25.74 ± 2.35 ns	659.10 ± 20.15 a
	KT	3.33 ± 0.16 ns	22.96 ± 0.42 a	1.11 ± 0.15 ns	22.14 ± 2.34 ns	612.60 ± 19.10 b
	UT	3.66 ± 0.22 ns	20.68 ± 0.42 b	0.92 ± 0.05 ns	23.18 ± 1.35 ns	481.80 ± 16.02 c
T		ns	*	ns	ns	*
Y		ns	ns	ns	ns	ns
T × Y		ns	ns	ns	ns	ns

Within each year, different letters indicate significant differences among treatments according to Tukey’s test (*p* < 0.05). The rows T, Y and Y × T refer to the overall effects of year, treatment and their interaction based on two-way ANOVA. * indicates a statistically significant effect (*p* < 0.05), whereas “ns” denotes a non-significant effect.

**Table 3:** Photosynthetic performance parameters (Fv’/Fm’, _Φ_PSII, qP, NPQ, and electron transport rate, ETR) measured in Nocera grapevines under different treatments (basalt powder (BPT), kaolin (KT), and untreated (UT)). Fv’/Fm’ represents the maximum efficiency of photosystem II (PSII) in light-adapted leaves; _Φ_PSII (effective quantum yield of PSII); qP (photochemical quenching); NPQ (non-photochemical quenching); ETR (electron transport rate). Values are expressed as mean ± standard deviation and refer to data collected over two growing seasons.

Year (Y)	Treatment (T)	Fv’/Fm’	_Φ_PSII	qP	NPQ	ETR µmol e^−^ m^−2^ s^−1^
2024	BPT	0.58 ± 0.02 a	0.12 ± 0.02 a	0.22 ± 0.02 a	2.28 ± 0.12 ns	97.5 ± 2.0 b
	KT	0.61 ± 0.01 a	0.14 ± 0.03 a	0.24 ± 0.01 a	2.28 ± 0.08 ns	111.0 ± 3.5 a
	UT	0.44 ± 0.10 b	0.03 ± 0.04 b	0.12 ± 0.02 b	1.93 ± 0.03 ns	66.0 ± 3.7 c
2025	BPT	0.60 ± 0.02 a	0.14 ± 0.02 a	0.24 ± 0.02 a	2.32 ± 0.14 ns	99.8 ± 2.3 b
	KT	0.63 ± 0.01 a	0.16 ± 0.03 a	0.26 ± 0.01 a	2.32 ± 0.10 ns	113.7 ± 3.9 a
	UT	0.46 ± 0.12 b	0.05 ± 0.04 b	0.14 ± 0.02 b	1.95 ± 0.03 ns	68.6 ± 3.9 c
T		*	*	*	ns	*
Y		ns	ns	ns	ns	ns
T × Y		ns	ns	ns	ns	ns

Within each year, different letters indicate significant differences among treatments according to Tukey’s test (*p* < 0.05). The rows T, Y and Y × T refer to the overall effects of year, treatment and their interaction based on two-way ANOVA. * indicates a statistically significant effect (*p* < 0.05), whereas “ns” denotes a non-significant effect.

**Table 4:** Effects of three foliar treatments—UT (untreated), KT (kaolin treatment), and BPT (basalt powder treatment)—on leaf gas exchange parameters and leaf microclimate. An = net photosynthetic rate; gs = stomatal conductance; Tr = transpiration rate; WUE = instantaneous water use efficiency (An/Tr); An/Ci = carboxylation efficiency; SPAD = chlorophyll content index; leaf temperature = canopy thermal status; VPD = vapor pressure deficit. Values are expressed as mean ± standard deviation and refer to data collected over two growing seasons.

Year (Y)	Treatment (T)	An (mol H_2_O m^−2^ s^−1^)	gs (mmol H_2_O m^−2^ s^−1^)	Tr (µmol CO_2_ mmol H_2_O^−1^)	WUE (µmol m^−2^ s^−1^)	An/Ci (µmol m^−2^ s^−1^)	SPAD	Leaf Temperature (°C)	VPD (kPa)
2024	BPT	7.82 ± 0.03 a	0.20 ± 0.05 a	2.30 ± 0.11 ns	3.38 ± 0.12 a	0.032 ± 0.03 a	28.50 ± 0.50 a	32.1 ± 0.6 b	2.48 ± 0.08 b
	KT	8.14 ± 0.09 a	0.19 ± 0.03 a	2.16 ± 0.10 ns	3.74 ± 0.17 a	0.033 ± 0.02 a	29.30 ± 0.70 a	31.2 ± 0.5 a	2.30 ± 0.07 a
	UT	5.58 ± 0.30 b	0.08 ± 0.04 b	2.00 ± 0.17 ns	2.78 ± 0.03 b	0.024 ± 0.01 b	24.70 ± 0.66 b	33.4 ± 0.7 c	2.70 ± 0.10 c
2025	BPT	7.84 ± 0.03 a	0.22 ± 0.05 a	2.32 ± 0.12 ns	3.40 ± 0.12 a	0.034 ± 0.03 a	28.76 ± 0.52 a	32.4 ± 0.7 b	2.52 ± 0.09 b
	KT	8.18 ± 0.09 a	0.21 ± 0.03 a	2.18 ± 0.10 ns	3.78 ± 0.17 a	0.035 ± 0.02 a	29.56 ± 0.74 a	31.5 ± 0.6 a	2.34 ± 0.08 a
	UT	5.66 ± 0.32 b	0.10 ± 0.04 b	2.02 ± 0.17 ns	2.80 ± 0.03 b	0.026 ± 0.01 b	24.92 ± 0.68 b	33.7 ± 0.8 c	2.74 ± 0.11 c
T		*	*	ns	*	*	*	*	*
Y		ns	ns	ns	ns	ns	ns	ns	ns
T × Y		ns	ns	ns	ns	ns	ns	ns	ns

Within each year, different letters indicate significant differences among treatments according to Tukey’s test (*p* < 0.05). The rows T, Y and Y × T refer to the overall effects of year, treatment and their interaction based on two-way ANOVA. * indicates a statistically significant effect (*p* < 0.05), whereas “ns” denotes a non-significant effect.

**Table 5:** Phenolic composition of grape berries from Nocera cv. subjected to three foliar treatments: KT (kaolin treatment), UT (untreated), and BPT (basalt powder treatment). The concentrations of individual phenolic compounds—including hydroxybenzoic acids (e.g., gallic acid, protocatechuic acid, vanillic acid), hydroxycinnamic acids (e.g., *p*-coumaric, *m*-coumaric, *o*-coumaric, caffeic, ferulic, and sinapic acids), flavan-3-ols (catechin, epicatechin, procyanidins), anthocyanidins (pelargonidin, delphinidin), flavonols (quercetin, kaempferol, myricetin, rutin, quercetin-3-β-D-glucoside), and other minor phenolics (chlorogenic acid, vicenin-2, eriocitrin, tocopherol, apigenin)—are presented below. Values are expressed as mean ± standard deviation for the 2024 growing season.

Phenolic Compound	KT	UT	BPT
Gallic acid	0.88 ± 0.09 a	0.66 ± 0.07 b	0.91 ± 0.10 a
Protocatechuic acid	0.43 ± 0.05 ns	0.42 ± 0.05	0.44 ± 0.05
Procyanidin B2	0.21 ± 0.03 ns	0.20 ± 0.03	0.21 ± 0.03
Syringic acid	0.42 ± 0.05 ns	0.43 ± 0.05	0.44 ± 0.05
*p*-Coumaric acid	0.01 ± 0.003 b	0.46 ± 0.06 a	0.04 ± 0.01 b
Pelargonidin	0.38 ± 0.04 ns	0.37 ± 0.04	0.39 ± 0.04
*m*-Coumaric acid	0.01 ± 0.003 ns	0.32 ± 0.05	0.17 ± 0.03
*o*-Coumaric acid	0.64 ± 0.08 a	0.26 ± 0.05 b	0.10 ± 0.03 c
Catechin	0.36 ± 0.05 a	0.07 ± 0.02 b	0.01 ± 0.003 b
Vanillic acid	0.04 ± 0.01 ns	0.04 ± 0.01	0.04 ± 0.01
Epicatechin	0.63 ± 0.09 a	0.01 ± 0.003 b	0.06 ± 0.02 b
Delphinidin	0.01 ± 0.003 ns	0.04 ± 0.01	0.02 ± 0.01
Trans-4-hydroxycinnamic acid	2.25 ± 0.20 ns	2.10 ± 0.18	2.20 ± 0.19
Sinapic acid	0.08 ± 0.01 ns	0.04 ± 0.01	0.04 ± 0.01
3-hydroxycinnamic acid	0.02 ± 0.004 ns	0.01 ± 0.003	0.02 ± 0.004
Myricetin	0.03 ± 0.01 ns	0.02 ± 0.01	0.05 ± 0.01
Luteolin	0.04 ± 0.01 ns	0.04 ± 0.01	0.05 ± 0.01
2,5-dihydroxybenzoic acid	0.03 ± 0.01 ns	0.03 ± 0.01	0.03 ± 0.01
Caffeic acid	0.02 ± 0.004 ns	0.01 ± 0.003	0.01 ± 0.003
Ellagic acid	0.07 ± 0.01 ns	0.06 ± 0.01	0.08 ± 0.01
Naringin	0.03 ± 0.01 ns	0.01 ± 0.003	0.01 ± 0.003
Quercetin	0.04 ± 0.01 ns	0.06 ± 0.01	0.07 ± 0.01
Kaempferol	0.03 ± 0.01 ns	0.02 ± 0.01	0.04 ± 0.01
Tocopherol	0.13 ± 0.02 ns	0.09 ± 0.02	0.05 ± 0.01
Procyanidin 1	0.04 ± 0.01 ns	0.05 ± 0.01	0.03 ± 0.01
Chlorogenic acid	0.03 ± 0.01 ns	0.03 ± 0.01	0.03 ± 0.01
Vicenin 2	0.06 ± 0.01 ns	0.11 ± 0.02	0.11 ± 0.02
Eriocitrin	0.13 ± 0.02 ns	0.14 ± 0.02	0.14 ± 0.02
Rutin	0.07 ± 0.01 ns	0.07 ± 0.01	0.07 ± 0.01
Quercetin-3-β-D-glucoside	0.02 ± 0.01 ns	0.01 ± 0.004	0.02 ± 0.01
Ferulic acid	0.02 ± 0.004 ns	0.02 ± 0.004	0.02 ± 0.004
Apigenin	0.85 ± 0.10 ns	0.74 ± 0.09	0.64 ± 0.08

Different letters within the same column indicate statistically significant differences among treatments according to Tukey’s test (*p* < 0.05). “ns” indicates no significant differences.

**Table 6:** Phenolic composition of grape berries from Nocera cv. subjected to three foliar treatments: KT (kaolin treatment), UT (untreated), and BPT (basalt powder treatment). The concentrations of individual phenolic compounds—including hydroxybenzoic acids (e.g., gallic acid, protocatechuic acid, vanillic acid), hydroxycinnamic acids (e.g., *p*-coumaric, *m*-coumaric, *o*-coumaric, caffeic, ferulic, and sinapic acids), flavan-3-ols (catechin, epicatechin, procyanidins), anthocyanidins (pelargonidin, delphinidin), flavonols (quercetin, kaempferol, myricetin, rutin, quercetin-3-β-D-glucoside), and other minor phenolics (chlorogenic acid, vicenin-2, eriocitrin, tocopherol, apigenin)—are presented below. Values are expressed as mean ± standard deviation for the 2025 growing season.

Phenolic Compound	KT	UT	BPT
Gallic acid	0.98 ± 0.08 a	0.78 ± 0.06 b	1.03 ± 0.09 a
Protocatechuic acid	0.49 ± 0.05 ns	0.50 ± 0.05	0.50 ± 0.05
Procyanidin B2	0.25 ± 0.03 ns	0.24 ± 0.03	0.25 ± 0.03
Syringic acid	0.48 ± 0.05 ns	0.47 ± 0.05	0.46 ± 0.05
*p*-Coumaric acid	0.01 ± 0.003 b	0.54 ± 0.06 a	0.06 ± 0.01 b
Pelargonidin	0.44 ± 0.04 ns	0.43 ± 0.04	0.45 ± 0.04
*m*-Coumaric acid	0.01 ± 0.003 ns	0.40 ± 0.05	0.23 ± 0.03
*o*-Coumaric acid	0.76 ± 0.07 a	0.34 ± 0.05 b	0.16 ± 0.03 c
Catechin	0.44 ± 0.05 a	0.11 ± 0.02 b	0.01 ± 0.003 b
Vanillic acid	0.06 ± 0.01 ns	0.06 ± 0.01	0.06 ± 0.01
Epicatechin	0.77 ± 0.08 a	0.01 ± 0.003 b	0.10 ± 0.02 b
Delphinidin	0.01 ± 0.003 ns	0.06 ± 0.01	0.04 ± 0.01
Trans-4-hydroxycinnamic acid	2.45 ± 0.22 ns	2.36 ± 0.19	2.40 ± 0.20
Sinapic acid	0.10 ± 0.01 ns	0.06 ± 0.01	0.06 ± 0.01
3-hydroxycinnamic acid	0.02 ± 0.004 ns	0.01 ± 0.003	0.04 ± 0.004
Myricetin	0.05 ± 0.01 ns	0.04 ± 0.01	0.07 ± 0.01
Luteolin	0.06 ± 0.01 ns	0.06 ± 0.01	0.07 ± 0.01
2,5-dihydroxybenzoic acid	0.03 ± 0.01 ns	0.03 ± 0.01	0.05 ± 0.01
Caffeic acid	0.02 ± 0.004 ns	0.01 ± 0.003	0.01 ± 0.003
Ellagic acid	0.09 ± 0.01 ns	0.08 ± 0.01	0.10 ± 0.01
Naringin	0.05 ± 0.01 ns	0.01 ± 0.003	0.01 ± 0.003
Quercetin	0.06 ± 0.01 ns	0.08 ± 0.01	0.09 ± 0.01
Kaempferol	0.05 ± 0.01 ns	0.04 ± 0.01	0.06 ± 0.01
Tocopherol	0.17 ± 0.02 ns	0.11 ± 0.02	0.07 ± 0.01
Procyanidin1	0.06 ± 0.01 ns	0.07 ± 0.01	0.05 ± 0.01
Chlorogenic acid	0.03 ± 0.01 ns	0.03 ± 0.01	0.03 ± 0.01
Vicenin2	0.08 ± 0.01 ns	0.13 ± 0.02	0.13 ± 0.02
Eriocitrin	0.13 ± 0.02 ns	0.14 ± 0.02	0.14 ± 0.02
Rutin	0.06 ± 0.01 ns	0.07 ± 0.01	0.07 ± 0.01
Quercetin-3-β-D-glucoside	0.01 ± 0.01 ns	0.01 ± 0.004	0.02 ± 0.01
Ferulic acid	0.01 ± 0.003 ns	0.02 ± 0.004	0.01 ± 0.004
Apigenin	0.81 ± 0.10 ns	0.71 ± 0.09	0.66 ± 0.08

Different letters within the same column indicate statistically significant differences among treatments according to Tukey’s test (*p* < 0.05). “ns” indicates no significant differences.

## Data Availability

The original contributions presented in this study are included in the article. Further inquiries can be directed to the corresponding author.
